# How to improve planetary health: Devising the ‘Planetary Health Approach’ from the biogeochemical flow perspectives

**DOI:** 10.7189/jogh.14.03014

**Published:** 2024-02-23

**Authors:** Yoshihiro Aoki, Ayumi Miyagi, Ayako Toyokawa, Shoko Misaka, Jin Yoshida, Abdelrahman M Makram, Abdelrahman Gamil Gad, Nguyen Tien Huy

**Affiliations:** 1 School of Tropical Medicine and Global Health, Nagasaki University, Nagasaki, Japan; 2 Coordination Office for Emergency Medicine and International Response, Acute and Clinical Care Center, Nagasaki University Hospital, Nagasaki, Japan; 3 Bureau of International Health Cooperation, National Centre for Global Health and Medicine, Tokyo, Japan; 4 Sendai City Public Health Centre, Sendai, Japan; 5 Healthcare Unit, Economic Research Institute for ASEAN and East Asia, Jakarta, Indonesia; 6 School of Public Health, Imperial College London, London, UK; 7 Faculty of Medicine, Ain Shams University, Cairo, Egypt; 8 Institute of Research and Development, Duy Tan University, Da Nang, Vietnam; 9 School of Medicine and Pharmacy, Duy Tan University, Da Nang, Vietnam

Planetary health is defined as ‘the health of human civilisation and the state of the natural systems on which it depends’ [1]; it is a relatively new concept and an unexplored field with little research in progress. It aims to provide and determine specific environmental limits to humanity within which they can safely operate. And with the interchangeable effects on planetary and environmental health, climate change harms human health, including decreased quality of life, increased disease burden, and elevated direct mortality rates [2].

In considering planetary health, nine boundaries of biological and physical processes are important for maintaining Earth’s health and function [3]. These are climate change, novel entities (which means ‘the growing awareness that toxic synthetic substances and other potentially systemic global risks exist’) [3], stratospheric ozone depletion, atmospheric aerosol loading, ocean acidification, biogeochemical flows, freshwater use, land-system change, and biosphere integrity. While each of these planetary boundaries is distinct from one other, major interplay and nonlinear relationships exist between them. Due to biogeochemical flows exceeding the high-risk zone, urgent countermeasures are needed. We explored the impact of nitrogen and phosphorous fertilisers on the environment and human health and offer solutions to mitigate these planetary health risks.

Biogeochemical flows include the circulation of various substances such as water, carbon, and sulphur. Among biogeochemical flow, the impact of nitrogen and phosphorus on the environment and human health has been emphasised, and they are also listed as targets in the nine boundaries in planetary health. The leading cause of excess nitrogen and phosphorus has been suggested to be fertilisers used by humans [3]. According to the World Bank, while chemical fertiliser use has been declining in some countries in recent years, the global trend has increased continuously [4]. However, it is estimated that the needed annual crops can be grown with 50% fewer fertilisers [5].

In the USA, excess nitrogen from fertiliser use leaches into groundwater, and an increased incidence of gastric cancer has been reported in farmers who rely on that water source [6]. Recently, *Karenia brevis* blooms, causing red tide through anthropogenic influence with nitrogen input have been reported in southwest Florida [7]. Indirect effects of residual fertiliser include the release of nitrogen oxide into the atmosphere through bacterial degradation, which accumulates and has a warming effect 300 times greater than carbon dioxide [8]. This contributes to increased flooding and droughts, food shortages, hurricanes, rising sea levels, and reduced habitable areas, posing significant human health risks.

Recently, China has succeeded in reducing fertiliser use while maintaining or increasing production [9]. The fertiliser usage should not be decreased unnecessarily but adjusted appropriately according to the land, climate, and target crops to improve efficiency in nitrogen use. In sub-Saharan Africa, despite high nitrogen use efficiency leading to soil mining, increasing fertiliser application can enhance crop production without expanding farmland [5]. The challenge is disseminating such facts and knowledge to raise awareness and put them into practice.

The Global Partnership on Nutrient Management seeks to address the environmental changes and health hazards caused by such biogeochemical flow-related nutrient issues. The Global Partnership on Nutrient Management, a sub-organisation of the United Nations, is an organisation that works with various stakeholders to improve nutrient management and provides a common platform worldwide, where it offers a variety of projects and research, as well as specific practices and policies, and promotes their effective use in the field. In practical case studies, the methodology shows how to gather the data, calculate nitrogen and phosphorus balance, and evaluate costs and benefits that local stakeholders can use in other settings.

**Figure 1:**
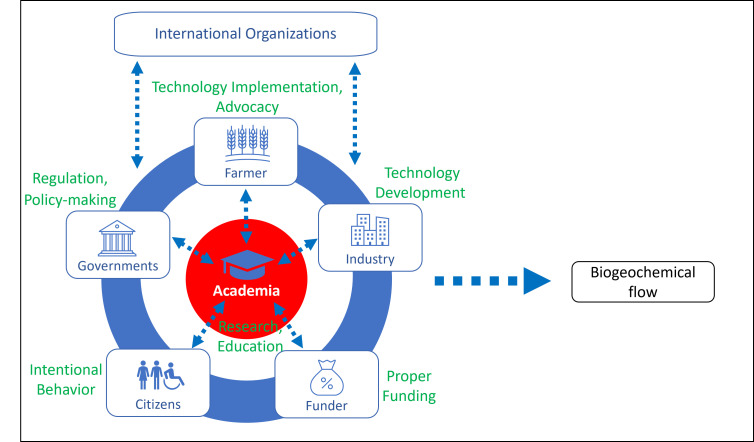
The concept figure of coordinated action by governments, academia, industry, farmers, funders, and citizens tackling environmental changes and human health hazards due to biogeochemical flow.

Environmental changes and human health hazards due to biogeochemical flow require coordinated action by governments, academia, industry, farmers, funders, and citizens (**Figure 1**). All stakeholders’ continuous dialogue to share their excellence and consider how to solve the issue together with horizontal connections is essential to improve biogeochemical flow. Governments have a role in developing regulations and laws, including adjusting fertiliser prices, providing financial assistance, or incentivising farmers. Since the needs of farmers vary from country to country in terms of region and climate, it may be necessary to establish a system for advocacy and active participation in policymaking by farmers to bring voices in the field to the government. Industry and farmers have attempted to improve nitrogen use efficiency by introducing new technologies, which should be supported with an adequate budget for further development and implementation. At this point, the European Union has a policy and a framework budgeted with six million euros on nitrogen and phosphorus pollution called ‘European Partnerships under Horizon Europe’ to achieve sustainable farming practices by 2050.

To achieve the transboundary approach, academia and research institutions should be at centre stage for clarifying in greater detail the effects of environmental destruction on human health by conducting collaborative research in many specialised fields. This research should be guided by governmental and international organisations to provide a broader and more global idea about the problem being addressed. Providing concrete advice to governments and industrial organisations on countermeasures would be essential. It is also required to calculate the appropriate amounts of fertilisers used in each region, communicate this information to the field, and raise farmers’ awareness to acquire the correct knowledge.

The concrete first step to be taken by academia is to raise awareness of the problem through education. An innovative educational curriculum should be developed to enable undergraduates to see horizontal connections and a broader perspective beyond the boundaries of the speciality. For example, it includes changing the medical curriculum to be more environment-oriented and to address biogeochemical health-related issues through specific subjects for university students. This makes future physicians more aware of the biogeochemical crisis and will enable them to diagnose and manage its related health issues [10]. It may also be helpful to foster researchers to conduct relevant collaborative research with a sustainable perspective. In addition, funding institutions and agencies should direct their research and education to biogeochemical flows, especially their effect on health, and make it a priority in the next few years to provide a realistic solution for the problem. The significance of biogeochemical flow-related health issues should be delivered correctly, leading to further fundraising for future research and education systems.

Furthermore, effective education can nurture deliberate actions towards responsible citizenship, which is essential in shaping a sustainable society. Citizens, the very essence of civilisation, have a pivotal role. Every choice, whether consuming organic produce, supporting sustainable practices, or advocating for green policies, shapes the narrative. Through collective consciousness, informed decisions, and empowered actions, citizens can steer the world toward a sustainable future. The Planetary Health Alliance has proposed an education framework to guide institutions, educators, and learners.

As supportive measures, establishing public-private partnerships and an evaluation system at the international level, such as the Global Partnership on Nutrient Management, may be crucial. It may also be important to consider focusing the updated version of the Sustainable Development Goals of Agriculture on sustainable, low-energy, and resilient agricultural methodologies. In this sense, we believe that ending hunger, preventing malnutrition, and doubling productivity cannot be achieved without considering rationing the use of fertilisers while maximising the outputs.

Rapid policy actions are still required while these solutions may be planned for the long term. We, therefore, propose these short-term actions. First, governments and researchers should investigate the discontinuities of yields and nitrogen pollution at international borders, as recently suggested by Wuepper et al. [11]. A second immediate solution for countries where farmers overuse fertilisers is to increase overall taxes on fertilisers and only provide the needed amount of fertilisers at a lower cost according to the academic recommendation or high-income countries. Moreover, precision farming and plant breeding technologies can be probable solutions to provide fertilisers to the field parts that only need enrichment [5]. Finally, as with public health/policy interventions, simultaneous high and low-agency interventions are needed. Therefore, educating farmers on sustainable practices, providing expert opinions through national media, and supporting these local communities and organisations can go a long way with national policy interventions.

Biogeochemical flow is critical when considering planetary health, and its changes are causing various health hazards, requiring enhanced countermeasures. Through collaborative research and innovative education, academia can devise efficient solutions to the overwhelming issue. Promoting the ‘Planetary Health Approach’ for each of the nine boundaries is necessary for the future.
